# *Dendrobium*×* falconerwardianum*, a natural hybrid of *D. wardianum* and *D. falconeri*

**DOI:** 10.7717/peerj.21153

**Published:** 2026-04-15

**Authors:** Qun Wang, Mengmeng Zhang, Sinuo Zhu, Yang Liu, Liu Xue, Yan Wang, Baoqiang Zheng

**Affiliations:** 1 Key Laboratory of Tree Breeding and Cultivation of National Forestry and Grassland Administration, Research Institute of Forestry, Chinese Academy of Forestry, Beijing, China; 2 Beijing Xishan Experimental Forest Farm Management Office, Beijing, China

**Keywords:** *Dendrobium* × *falconerwardianum*, *Dendrobium wardianum*, *Dendrobium falconeri*, Morphology, Phylogeny

## Abstract

We discovered an unknown * Dendrobium* taxon from the northwestern region of Tengchong City, Yunnan Province, China, which exhibits characteristics intermediate between *Dendrobium wardianum* and *Dendrobium falconeri*. Morphological analysis indicated that this taxon displays a mosaic of morphological characters derived from sympatric * D. wardianum* and *D. falconeri* with overlapping flowering periods: its vegetative organs (cylindrical stem, inconspicuous nodes, narrowly oblong leaves) are consistent with *D. wardianum*; the floral sepal shape (ovate-lanceolate) and wavy lip margins are similar to *D. falconeri*; while the two dark purple patches at the base of the lip are inherited from *D. wardianum*. It is named * Dendrobium* × *falconerwardianum*. Molecular biological analysis based on nuclear markers (internal transcribed spacer, ITS) and plastid markers (*ribulose-1,5-bisphosphate carboxylase*/*oxygenase large subunit*, *rbcL*) also indicated that * D.* × * falconerwardianum* is a natural hybrid of * D. wardianum* and * D. falconeri*. Based on combined morphological and molecular analyses, it is concluded that this taxon represents a natural hybrid of * D. wardianum* and * D. falconeri*. This discovery not only enriches the diversity of the genus * Dendrobium*, but also provides new insights into the evolutionary dynamics and gene flow barriers within the genus.

## Introduction

*Dendrobium* Swartz (1799) is one of the largest genera in the Orchidaceae, widely distributed across tropical and subtropical regions from Asia to Oceania (Odyuo, Deori & Daimary, 2017; Zheng et al., 2020). The genus is characterized by lateral inflorescences, lateral sepals adnate to the column forming a mentum, and four exposed pollinia (Xu et al., 2014; Zheng et al., 2025). While many *Dendrobium* species are renowned for their medicinal and ornamental value, their commercial demand has driven over-collection and habitat destruction, severely threatening wild populations (Subedi et al., 2013; Feng et al., 2015; Phelps, 2015; Gale et al., 2019). Consequently, accurate species identification is critical not only for conservation but also for assessing biodiversity and evolutionary processes within the genus.

The taxonomy of *Dendrobium* is complex due to morphological diversity and plasticity (Tian et al., 2017). Traditional identification relies on phenotypic traits, which are often influenced by environmental conditions. Furthermore, in the absence of flowering stages, species differentiation becomes extremely difficult (Feng et al., 2015). These challenges make molecular biological methods indispensable. The internal transcribed spacer (ITS), a biparentally inherited non-coding region with low functional constraints, exhibits both conservation and variability suitable for distinguishing species (Feng et al., 2015; Qin et al., 2017). In the context of hybrid identification, ITS provides nuclear evidence of biparental contributions. Plastid DNA sequences, such as *ribulose-1,5-bisphosphate carboxylase*/*oxygenase large subunit* (*rbcL*), serve as a complementary resource due to their maternal inheritance in most orchids (Daniell et al., 2021; Yang et al., 2023). The combination of biparental ITS and maternally inherited plastid markers allows for the unambiguous determination of hybrid origin by revealing the specific maternal parent, which is often difficult to infer from morphology alone. The integration of these markers has significantly advanced our understanding of *Dendrobium* phylogeny, leading to the discovery of numerous new species and varieties, such as *Dendrobium wenshanense* Q Xu, YB Luo & ZJ Liu (Xu et al., 2014), *Dendrobium longlingense* Q Xu, YB Luo & ZJ Liu (Xu et al., 2014), *Dendrobium maguanense* Q Xu & ZJ Liu (Xu et al., 2017), *Dendrobium bannaense* YQ Tian & YB Huang (Tian et al., 2017), *Dendrobium tuensangense* (Odyuo, Deori & Daimary, 2017), *Dendrobium jinghuanum BQ Zheng & Y Wang* (Zheng et al., 2020), *Dendrobium nobile* var. *cooksonianum* Rchb.f. (Liu et al., 2014), and *Dendrobium moniliforme* var. *hongbinii* HB Yang & BQ Zheng (Zheng et al., 2025).

Although natural hybridization is common in the Orchidaceae, naturally occurring hybrids in *Dendrobium* with clearly identified parental species are rarely documented. For instance, *Cypripedium* × *microsaccos* is a hybrid between *Cypripedium calceolus* and *Cypripedium shanxiense*, and *C.* × *ventricosum* is a hybrid between *C. calceolus* and *Cypripedium macrantho* (Wang et al., 2025a; Wang et al., 2025b). Investigating these hybridization events is essential for understanding gene flow and speciation mechanisms in the genus. Recently, during an expedition in the northwestern region of Tengchong City, Yunnan Province, China, we discovered a distinct, unknown *Dendrobium* taxon growing on the trunks of trees in dense forests. Its morphological characteristics appear intermediate between two sympatric species with overlapping flowering periods: *Dendrobium wardianum* and *Dendrobium falconeri*. This observation raises a key scientific question: does this intermediate taxon represent a natural hybridization event between these two species? We hypothesize that this unknown taxon is a natural hybrid of *D. wardianum* and *D. falconeri*. To test this, we integrate morphological comparisons with molecular phylogenetic analysis to reveal its true identity and provide evidence for the evolutionary dynamics of *Dendrobium*.

## Materials & Methods

### Morphological analysis

The newly recorded nothospecies was collected from the northwestern region of Tengchong City, Yunnan Province, China. The morphological characteristics of the flowers of the newly recorded nothospecies in full bloom were observed and photographed using a digital camera (Canon EOS R50). The type specimen was preserved in the Herbarium of the Research Institute of Forestry, Chinese Academy of Forestry (RIF), with the specimen number HYBRID3.

### Acquisition of DNA sequence

The total DNA of the newly recorded nothospecies was extracted using the Rapid Plant Genomic DNA Isolation Kit (Sangon Biotech Co., Ltd., Shanghai, China). The primers were synthesized by Ruibo Xingke Biotechnology Co., Ltd. (Beijing, China) based on the information provided by Xiang et al. (2013); the primer sequences are shown in Table S1. Using these synthesized primers, the ITS and *rbcL* sequences of the newly recorded nothospecies were amplified, and the sequencing was performed by Sangon Biotech Co., Ltd. (Shanghai, China). The newly generated ITS and *rbcL* sequences of the newly recorded nothospecies were submitted to GenBank (https://submit.ncbi.nlm.nih.gov/subs/genbank/; Table 1). The ITS and *rbcL* sequences of other *Dendrobium*-related species and outgroups were obtained from GenBank *via* accession numbers (Table 1).

**Table 1:** The list of GenBank accession numbers for the ITS and *rbcL* sequences used in phylogenetic analysis.

**Species**	**ITS**	***rbcL***	**Species**	**ITS**	***rbcL***
*D.*×*falconerwardianum*	PV646510^*^	PV660428^*^	*D. infundibulum*	KF143477	KF177618
*D. aduncum*	KF143428	KF177571	*D. jenkinsii*	KF143478	KF177619
*D. aggregatum*	KF143429	KF177572	*D. loddigesii*	KF143481	KF177622
*D. aphyllum*	PV643874	PV660426	*D. lohohense*	KF143482	KF177623
*D. bellatulum*	KF143431	KF177574	*D. longicornu*	KF143483	KF177624
*D. bensoniae*	PP516854	NC072269	*D. menglaense*	KF143486	KF177627
*D. brymerianum*	KF143432	KF177575	*D. minutiflorum*	KF143487	KF177628
*D. capillipes*	KF143433	KF177576	*D. moniliforme*	KF143489	KF177630
*D. cariniferum*	KF143435	KF177579	*D. moschatum*	KF143492	KF177633
*D. catenatum*	KF143438	KF177583	*D. nobile*	KF143493	KF177634
*D. christyanum*	KF143442	KF177585	*D. okinawense*	KF143495	KF177636
*D. chrysanthum*	KF143443	KF177586	*D. pendulum*	KF143496	KF177637
*D. chrysotoxum*	KF143444	KF177587	*D. porphyrochilum*	KF143500	KF177641
*D. crepidatum*	KF143446	KF177589	*D. primulinum*	KF143499	KF177640
*D. crystallinum*	KF143447	KF177590	*D. pulchellum*	KF143503	KF177644
*D. denneanum*	KF143448	KF177591	*D. ruckeri*	KF143504	KF177645
*D. densiflorum*	KF143451	KF177594	*D. salaccense*	KF143506	KF177647
*D. denudans*	KF143452	KF177595	*D. scoriarum*	KF143508	KF177649
*D. devonianum*	KF143453	KF177596	*D. senile*	KF143509	KF177650
*D. dixanthum*	KF143454	KF177597	*D. signatum*	PP758186	PV015130
*D. ellipsophyllum*	KF143455	KF177598	*D. sinense*	KF143510	KF177651
*D. exile*	KF143456	KF177599	*D. spatella*	KF143512	KF177653
*D. falconeri*	KF143458	KF177674	*D. strongylanthum*	KF143514	KF177655
*D. fanjingshanense*	KF143459	KF177601	*D. stuposum*	KF143516	KF177657
*D. fimbriatum*	KF143461	KF177603	*D. sulcatum*	KF143517	KF177658
*D. findlayanum*	KF143462	KF177604	*D. thyrsiflorum*	KF143519	KF177659
*D. goldschmidtianum*	KF143463	KF177605	*D. transparens*	KF143520	KF177660
*D. gratiosissimum*	KF143464	KF177606	*D. trigonopus*	KF143521	KF177661
*D. hainanense*	KF143466	KF177607	*D. wangliangii*	KF143524	KF177664
*D. hancockii*	KF143467	KF177608	*D. wardianum*	PV643878	PV660427
*D. harveyanum*	KF143468	KF177609	*D. wattii*	KF143525	KF177665
*D. henanense*	KF143469	KF177610	*D. wilsonii*	KF143526	KF177666
*D. henryi*	KF143470	KF177611	*D. xichouense*	KF143527	KF177667
*D. hercoglossum*	KF143471	KF177612	*D. zhenyuanense*	KF143528	KF177668
*D. heterocarpum*	KF143473	KF177614	*B. disciflorum*	KY966444	KM924502
*D. hookerianum*	KF143474	KF177615	*B. oblongum*	KY966465	KM924498
*D. huoshanense*	KF143476	KF177617			

**Notes.**

* indicates sequences obtained in this study.

### Phylogenetic analysis

Phylogenetic analysis was conducted using PhyloSuite v1.2.2 for the newly recorded nothospecies, 70 known *Dendrobium* species, and 2 *Bulbophyllum* species *via* the Maximum Likelihood (ML) method (Zhang et al., 2020). Sequence alignment was performed using MAFFT (Katoh, Rozewicki & Yamada, 2019), and the aligned sequences were trimmed using Gblocks (Talavera & Castresana, 2007). ModelFinder was employed to determine the best-fit model (Kalyaanamoorthy et al., 2017). All of the aforementioned steps were performed using the default parameters of the software. Finally, the ML phylogenetic tree was constructed using IQ-TREE (Minh et al., 2020). The attributes, composition, and best-fit model of the dataset used to construct the phylogenetic tree are shown in Table 2. The phylogenetic tree was visualized using ChiPlot (https://www.chiplot.online/) (Xie et al., 2023).

**Table 2:** Statistics and best-fit models from the phylogenetic analyses.

**Information**	**ITS**	***rbcL***
No. taxa	73	73
Aligned length	651	1,246
No. variable characters	429	95
No. parsimony-informative characters	333	56
Tree length	1,829	140
Consistency index (CI)	0.411	0.743
Retention index (RI)	0.667	0.866
Best-fit models	SYM+I+G4	K81+R2

### Nomenclatural acts

The electronic version of this article in Portable Document Format (PDF) will represent a published work according to the International Code of Nomenclature for algae, fungi, and plants (ICN), and hence the new names contained in the electronic version are effectively published under that Code from the electronic edition alone. In addition, new names contained in this work which have been issued with identifiers by IPNI will eventually be made available to the Global Names Index. The IPNI LSIDs can be resolved and the associated information viewed through any standard web browser by appending the LSID contained in this publication to the prefix “http://ipni.org/”. The online version of this work is archived and available from the following digital repositories: PeerJ, PubMed Central SCIE, and CLOCKSS.

## Results

### Evidence for hybrid origin

**Figure 1:**
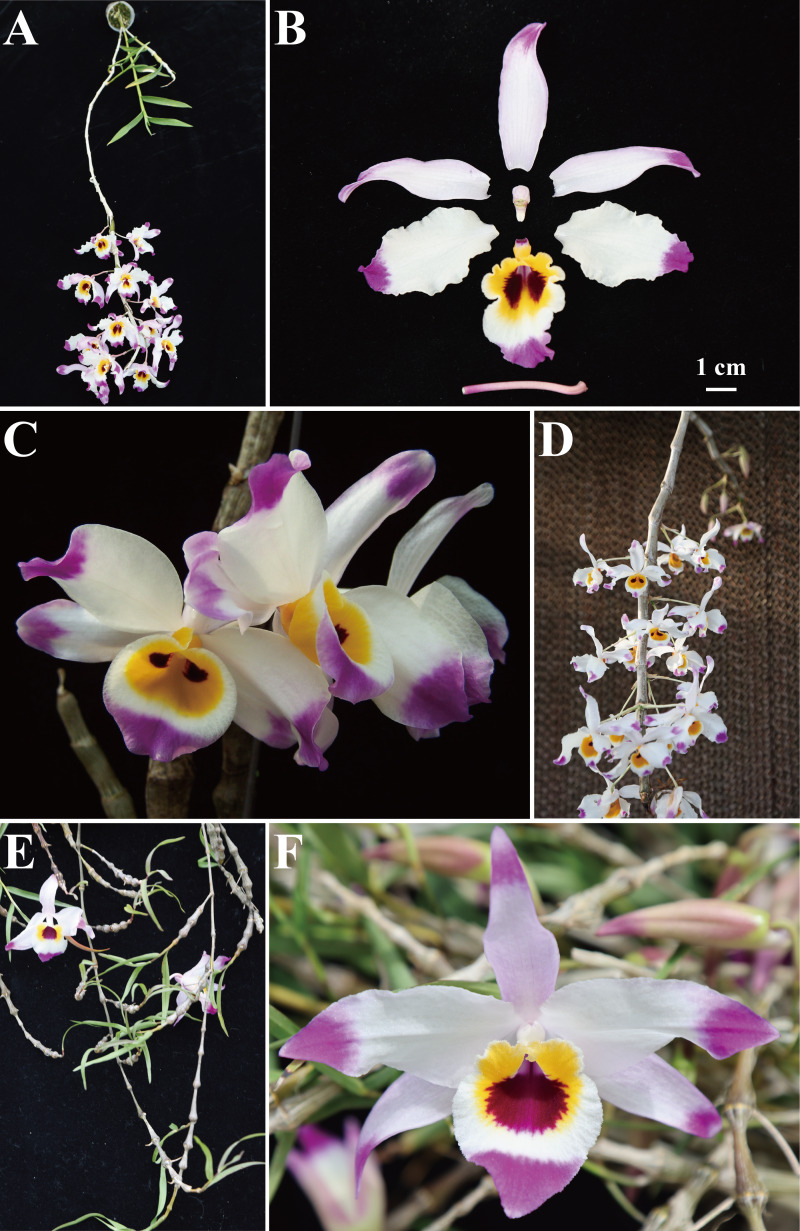
Comparison of *D.*×*falconerwardianum* with *D. wardianum* and *D. falconeri*. (A) Whole plant of *D.* × *falconerwardianum*; (B) Floral dissection of *D.* ×* falconerwardianum*; (C) Front view of the whole flower of *D. wardianum*; (D) Whole plant of *D. wardianum*; (E) Whole plant of *D. falconeri*; (F) Front view of the whole flower of *D. falconeri*.

Based on detailed morphological comparisons with data from the Plant Science Data Center (https://www.plantplus.cn/cn), we infer that the presumed parents of this unknown taxon are *D. wardianum* and *D. falconeri*. Notably, *D.* × *falconerwardianum* exhibits a mosaic of intermediate characteristics specific to both putative parents, supporting its status as a natural hybrid. Specifically, in terms of vegetative morphology, *D.* × *falconerwardianum* and *D. wardianum* share a cylindrical stem with inconspicuous nodes and narrowly oblong leaves, whereas *D. falconeri* has a moniliform stem with distinct nodes and narrowly lanceolate leaves (Figs. 1A–1F; Table 3). Regarding floral morphology, the hybrid displays intermediate traits: while its sepals are ovate-lanceolate as in *D. falconeri*, its lip margins are wavy—a feature also observed in *D. falconeri* but distinct from the smooth margins of *D. wardianum*. However, the lip of the hybrid possesses two dark purple patches in the basal region, a specific characteristic inherited from *D. wardianum*, which contrasts with the single patch found in *D. falconeri* (Figs. 1A–1F; Table 3). These combined morphological features—specifically the stem shape from *D. wardianum*, the sepal shape from *D. falconeri*, and the intermediate lip pattern—strongly indicate that the discovered taxon is a natural hybrid between these two *Dendrobium* species, rather than a previously described species undocumented in the region. Detailed comparisons are shown in Table 3.

**Figure 2:**
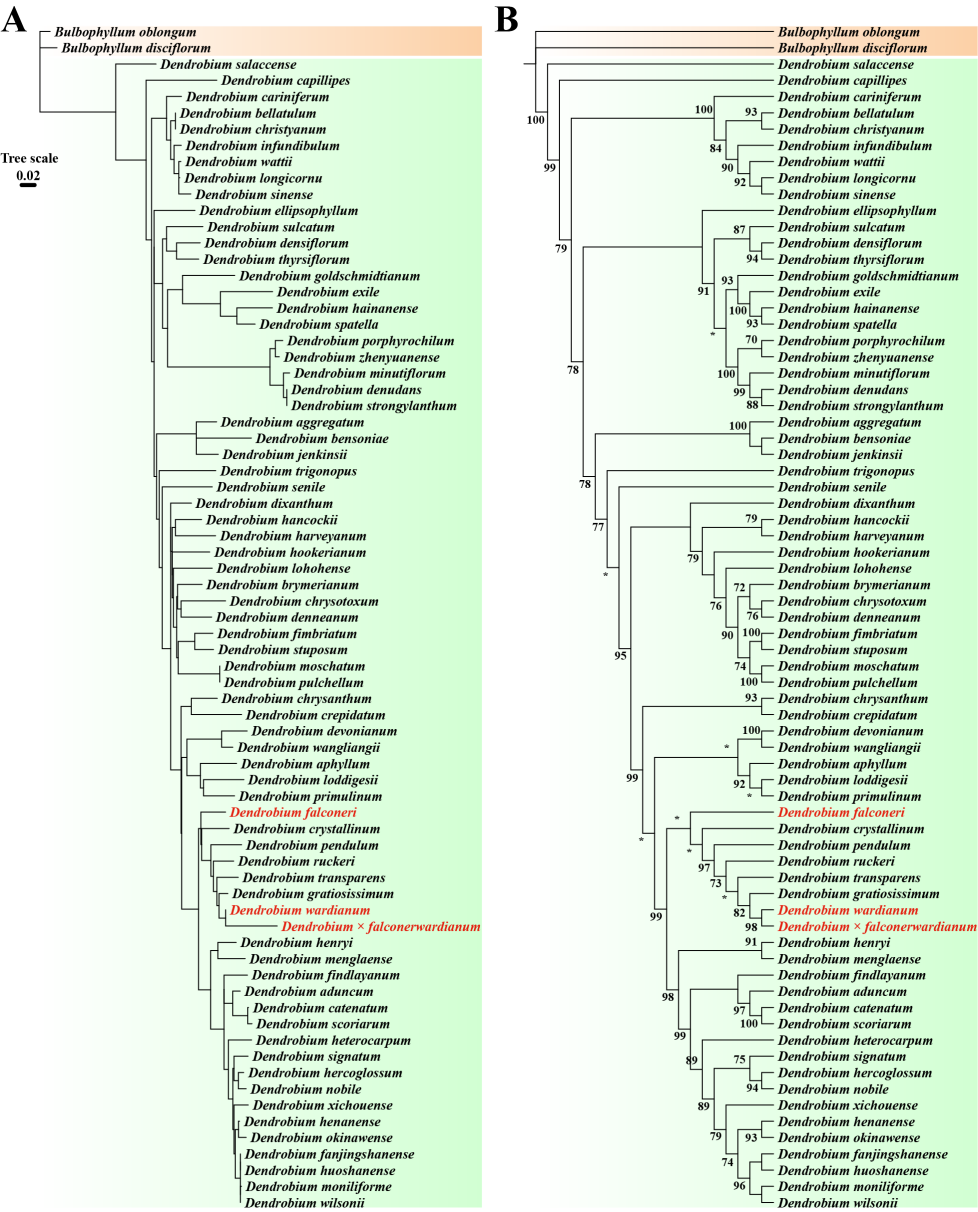
ML phylogenetic tree based on ITS sequences constructed under the SYM+I+G4 model. (A) ML phylogenetic tree retaining genetic distances; (B) ML phylogenetic tree ignoring genetic distances. Numbers on the nodes indicate BP_ML. “*” indicates nodes with support rates lower than 70% in the ML analysis.

**Figure 3:**
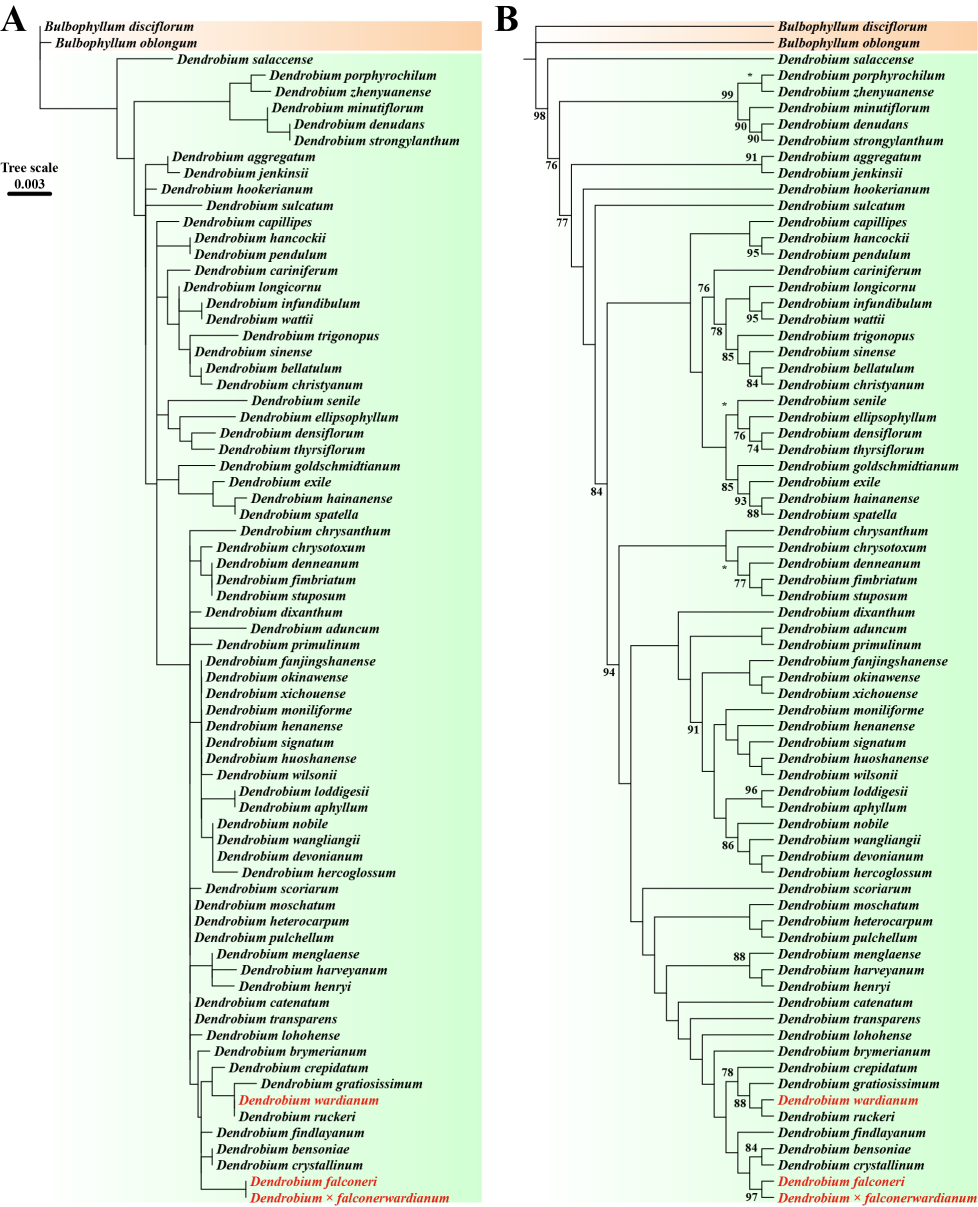
ML phylogenetic tree based on *rbcL* sequences constructed under the K81+R2 model. (A) ML phylogenetic tree retaining genetic distances; (B) ML phylogenetic tree ignoring genetic distances. Numbers on the nodes indicate BP_ML_. “*” indicates nodes with support rates lower than 70% in the ML analysis.

Successful amplification of ITS and *rbcL* sequences yielded fragment lengths of 874 bp and 1,262 bp, respectively (Fig. S1). Phylogenetic analysis based on the biparentally inherited ITS revealed that *D.* × *falconerwardianum* is sister to *D. wardianum*, which suggests that *D. wardianum* contributes the nuclear genome (Maximum likelihood bootstrap percentages (BP_ML_) = 98; Figs. 2A, 2B). Meanwhile, phylogenetic analysis based on the maternally inherited *rbcL* showed that *D.* × *falconerwardianum* is sister to *D. falconeri*, implying that it is the maternal parent (BP_ML_ = 97; Figs. 3A, 3B). These molecular discrepancies unequivocally support the hybrid origin of this taxon.

**Table 3:** Morphological comparison of D*.*×* falconerwardianum*, *D. wardianum*, and *D. falconeri*.

Trait	*D.*×*falconerwardianum*	*D. wardianum*	*D. falconeri*
Stem	Cylindrical stem, inconspicuous nodes	Cylindrical stem, inconspicuous nodes	Moniliform stem, distinct nodes
Leaf	Narrowly oblong leaves	Narrowly oblong leaves	Narrowly lanceolate leaves
Sepal	Ovate-lanceolate sepals	Elliptic sepals	Ovate-lanceolate sepals
Petal	Petals with wavy margins	Petals with smooth margins	Petals with smooth margins
Lip	Lip with wavy margins and two dark purple patches	Smooth lip with smooth margins and two dark purple patches	Lip with wavy margins and one dark purple patch

### Taxonomic treatment

*Dendrobium* × *falconerwardianum* BQ Zheng & Q Wang, nothosp. nov. Figs. 1A, 1B

urn:lsid:ipni.org:names: 77377654-1

**Chinese name**: “zhū qiào shí hú” (珠鞘石斛)

**Type:** CHINA. Yunnan Province, Tengchong City, NW region, ca. 1,200 m, epiphytes on tree trunks in broad-leaved forests, 26 Apr 2019, *Bao-Qiang Zheng* s.n. (holotype: CAF HYBRID3!).

**Diagnosis:** *D.* × *falconerwardianum* is a natural hybrid between *D. wardianum* and *D. falconeri*, exhibiting a mosaic of parental characters. It differs from *D. falconeri* by the cylindrical stem with inconspicuous nodes and narrowly oblong leaves. It is distinguished from *D. wardianum* by the ovate-lanceolate sepals and lip with wavy margins. Additionally, the lip base possesses two dark purple patches (inherited from *D. wardianum*), contrasting with the single patch found in *D. falconeri*.

**Description:** Epiphytic herbs. Stems obliquely erect or pendulous, fleshy, cylindrical, 25–60 cm long, 6–11 mm in diameter, branched; internodes numerous, slightly swollen, clavate, 2–4 cm long, yellowish with dirty black when dried. Leaves thin-coriaceous, distichous, narrowly oblong, 4–12 cm long, 1–1.5 cm wide, apex acute, base sheathing; leaf sheaths papery, white, tightly clasping the stem, mouth often opening when dry. Inflorescences terminal, from upper nodes, 1–3-flowered; peduncle and ovary slender. Flowers opening widely. Dorsal sepal ovate-lanceolate; lateral sepals ovate-lanceolate, slightly larger than dorsal sepal, forming a mentum with the column foot. Petals elliptic-oblong, margins undulate. Lip 3-lobed, elliptic or broadly ovate in outline, margins undulate, adaxial surface with two dark purple patches near base. Column short, with a distinct foot; anther cap subglobose; pollinia 4, in two pairs.

**Phenology:** Flowering from April to May.

**Etymology:** The epithet “*falconerwardianum*” is derived from the combination of the specific epithets of the parent species *D. falconeri* and *D. wardianum*, reflecting its hybrid nature.

**Distribution and habitat:** *D.* × *falconerwardianum* was collected from the northwestern region of Tengchong City, Yunnan Province, China. It grows epiphytically on tree trunks in broad-leaved forests at an altitude of approximately 1,200 m.

**Conservation status:** Only five individuals (ramets) were found at the type locality, which is situated in a montane forest habitat. To date, the number of mature individuals of this orchid is less than 10, and no further data on its population status have been obtained. Both putative parental species, *D. wardianum* and *D. falconeri*, are present in this locality, and their flowering phenologies overlap, providing conditions for natural hybridization. Currently, this hybrid is known only from this type locality; no other sympatric areas where hybridization could occur have been reported. Molecular marker analysis identified *D. falconeri* as the maternal parent and *D. wardianum* as the paternal parent.

## Discussions

*Dendrobium* exhibits complex intra- and interspecific morphological variations, often making taxonomic identification difficult, particularly when carefully distinguishing between natural hybrids and ecotypes arising from environmental adaptation (Xiang et al., 2013; Liu et al., 2014; Qi, Huang & Zhang, 2020; Zheng et al., 2020; Zheng et al., 2025). Here we report that *D.* × *falconerwardianum* displayed a mosaic of intermediate characteristics of parental specificity in key traits such as the stem, leaf, sepal, petal, and lip. This stable, discontinuous combination of traits reflects the recombination of allogeneic genomes, which is distinct from the continuous gradual variation of singular quantitative traits induced by environmental stress (*i.e.,* phenotypic plasticity) typically observed in ecotypes (Bennett et al., 2023). Molecular biological evidence further supports this conclusion: the analysis of the matrilineally inherited *rbcL* sequence clustered the taxon with the maternal parent, confirming its maternal origin; meanwhile, the nuclear ITS sequence confirmed the origin of the other parent. Although hybrids are theoretically expected to possess ITS sequences from both parents, the hybrid in this study only showed affinity to *D. wardianum*. Such uniparental bias (non-additivity) of ITS sequences is not uncommon in plant hybrids. A possible explanation is that concerted evolution of nuclear ribosomal DNA occurred during subsequent evolution or backcrossing, wherein the *D. falconeri*-derived ITS sequences were homogenized or replaced by the *D. wardianum* type, eventually leading to the complete homogenization of ITS sequences in the hybrid population. Based on combined evidence from morphological intermediacy and molecular phylogeny, we exclude the possibility of it being an ecotype and confirm *D.* × *falconerwardianum* as a natural hybrid between *D. wardianum* and *D. falconeri*.

This finding not only confirms the taxonomic status of this hybrid but also provides new insights into the evolutionary dynamics and gene flow within *Dendrobium*. In terms of breeding, the hybrid demonstrates a successful combination of parental traits, suggesting the potential presence of valuable introgression in its genome (Kaneko & Bang, 2014; Adhikari et al., 2023). This provides parental material for the future utilization of heterosis or the selection of new varieties with specific floral morphology. Regarding conservation, the clear identification of this natural hybrid is critical for conservation strategies; it aids in monitoring changes in genetic purity within wild populations and prevents the loss of unique genotypes of rare parents caused by frequent hybridization (Scriber, 2013). Despite this, the study has certain limitations. Due to the discovery of only a limited number of wild individuals, the small sample size and the lack of controlled cultivation experiments under different environmental conditions restrict a comprehensive assessment of the phenotypic stability of this hybrid across various environments. Future research is needed to expand the sampling range, conduct artificial hybridization verification, and perform multi-environmental phenotypic observations to further verify the genetic stability and adaptive potential of this hybrid.

## Conclusions

In summary, this study provides comprehensive evidence from both morphology and molecular phylogenetics to identify a previously unknown *Dendrobium* taxon from Tengchong, Yunnan, as a natural hybrid between *D. wardianum* and *D. falconeri*. We formally designate this taxon as *D.* × *falconerwardianum*. The morphological analysis reveals a distinct mosaic of parental traits—specifically the vegetative organs of *D. wardianum* combined with the floral features of *D. falconeri*—which effectively distinguishes it from environmentally induced ecotypes or phenotypic plasticity. Furthermore, molecular evidence from nuclear ITS and plastid *rbcL* markers confirms the biparental origin of this hybrid, identifying *D. wardianum* as the maternal parent. The confirmation of *D.* × *falconerwardianum* not only enriches the known biodiversity of the genus *Dendrobium* but also contributes to a deeper understanding of evolutionary dynamics, including gene flow and speciation mechanisms. From a practical standpoint, this discovery offers valuable genetic resources for future breeding programs focused on heterosis and novel trait selection, while also providing critical data for formulating effective conservation strategies to monitor and protect genetic purity in wild populations. Future research involving expanded sampling, artificial hybridization verification, and multi-environmental cultivation will be essential to further assess the genetic stability and adaptive potential of this hybrid.

##  Supplemental Information

10.7717/peerj.21153/supp-1 Supplemental Information 1 PCR amplification results of ITS and *rbcL* sequences (A) Amplification results of ITS; (B) Amplification results of *rbcL*.

10.7717/peerj.21153/supp-2 Supplemental Information 2 ITS and *rbcL* primer sequences

## Additional Information and Declarations

### Data Availability

The following information was supplied regarding data availability: The DNA/RNA sequences are available at Genbank: PV646510 and PV660428.

### New Species Registration

The following information was supplied regarding the registration of a newly described species: *Dendrobium* × *falconerwardianum* B.Q. Zheng & Q. Wang
