# Supplementary figures and images for: Dendrobium× falconerwardianum, a natural hybrid of D. wardianum and D. falconeri
Source: PeerJ. 2026 Apr 15;14:e21153. doi: 10.7717/peerj.21153 (PMC13091582; doi:10.7717/peerj.21153)

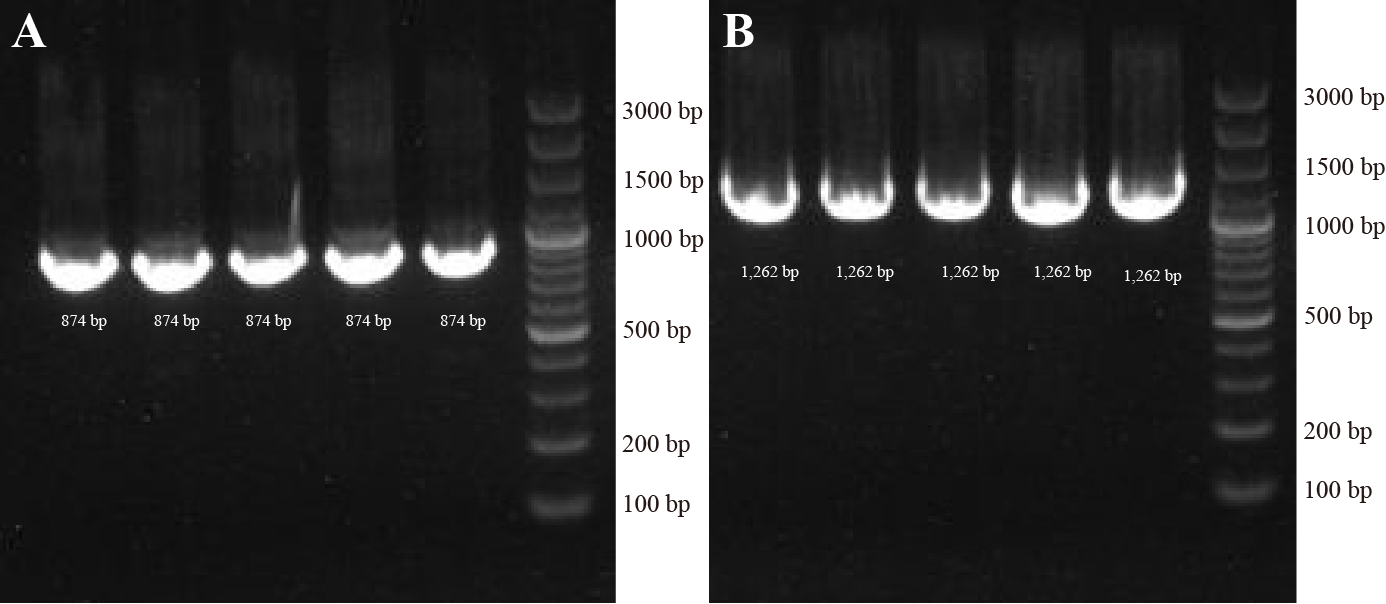

Supplement: Supplemental Information 1 — (A) Amplification results of ITS; (B) Amplification results of rbcL. [file peerj-14-21153-s001.png]
