# Supplementary material for: Dendrobium× falconerwardianum, a natural hybrid of D. wardianum and D. falconeri
Source: PeerJ. 2026 Apr 15;14:e21153. doi: 10.7717/peerj.21153 (PMC13091582; doi:10.7717/peerj.21153)
Supplement: Supplemental Information 2 [file peerj-14-21153-s002.docx]

**Table S1.** ITS and *rbcL* primer sequences

| DNA regions | Forward primer sequence (5'-3') | Reverse primer sequence (5'-3') |
| --- | --- | --- |
| ITS | ACGAATTCATGGTCCGGTGAAGTGTTCG | TAGAATTCCCCGGTTCGCTCGCCGTTAC |
| *rbcL* | ATGTCACCACAAACAGAAAC | CTTCACAAGCAGCAGCTAGTTC |
